# Identifying Loci Associated With Bovine Corona Virus Infection and Bovine Respiratory Disease in Dairy and Feedlot Cattle

**DOI:** 10.3389/fvets.2021.679074

**Published:** 2021-08-02

**Authors:** Jennifer N. Kiser, Holly L. Neibergs

**Affiliations:** Department of Animal Sciences, Washington State University, Pullman, WA, United States

**Keywords:** bovine coronavirus, bovine respiratory disease, cattle, genome-wide association analysis, loci

## Abstract

Bovine coronavirus (BCoV) is associated with respiratory and enteric infections in both dairy and beef cattle worldwide. It is also one of a complex of pathogens associated with bovine respiratory disease (BRD), which affects millions of cattle annually. The objectives of this study were to identify loci and heritability estimates associated with BCoV infection and BRD in dairy calves and feedlot cattle. Dairy calves from California (*n* = 1,938) and New Mexico (*n* = 647) and feedlot cattle from Colorado (*n* = 915) and Washington (*n* = 934) were tested for the presence of BCoV when classified as BRD cases or controls following the McGuirk scoring system. Two comparisons associated with BCoV were investigated: (1) cattle positive for BCoV (BCoV^+^) were compared to cattle negative for BCoV (BCoV^−^) and (2) cattle positive for BCoV and affected with BRD (BCoV^+^BRD^+^) were compared to cattle negative for BCoV and BRD (BCoV^−^BRD^−^). The Illumina BovineHD BeadChip was used for genotyping, and genome-wide association analyses (GWAA) were performed using EMMAX (efficient mixed-model association eXpedited). The GWAA for BCoV^+^ identified 51 loci (*p* < 1 × 10^−5^; 24 feedlot, 16 dairy, 11 combined) associated with infection with BCoV. Three loci were associated with BCoV^+^ across populations. Heritability estimates for BCoV^+^ were 0.01 for dairy, 0.11 for feedlot cattle, and 0.03 for the combined population. For BCoV^+^BRD^+^, 80 loci (*p* < 1 × 10^−5^; 26 feedlot, 25 dairy, 29 combined) were associated including 14 loci across populations. Heritability estimates for BCoV^+^BRD^+^ were 0.003 for dairy, 0.44 for feedlot cattle, and 0.07 for the combined population. Several positional candidate genes associated with BCoV and BRD in this study have been associated with other coronaviruses and respiratory infections in humans and mice. These results suggest that selection may reduce susceptibility to BCoV infection and BRD in cattle.

## Introduction

The coronaviridae family, from the Nidoviruse order, consists of enveloped, positive-stranded RNA viruses with some of the largest viral genomes known among all RNA viruses. This family is often split into groups (1–3) based on serological and genetic similarities between the viruses (1). Bovine coronavirus (BCoV) is one of the common viral pathogens associated with bovine respiratory disease (BRD) and is a group 2 coronavirus along with human coronavirus OC43, murine hepatitis virus, canine respiratory coronavirus, equine coronavirus, and rat sialodacryadenitis virus (1). Several group 2 coronaviruses, including BCoV, have a unique surface glycoprotein, hemagglutinin-esterase (HE), and a spike protein. The expression of the HE glycoprotein has been linked to enhanced virulence of some group 2 coronaviruses (2). In cattle, Popova and Zhang (3) determined that the presence of the spike protein alone, but not the HE protein by itself, was adequate for viral infection in host cells. However, HE is involved in viral attachment of host cells to some extent (4). Interestingly, monoclonal antibodies against HE were able to neutralize BCoV infection *in vitro* (5) and protect the cattle intestinal epithelium from viral infection *in vivo* (6). This suggests that the HE protein may have a role in inducing a protective response during BCoV infection. The spike protein of coronaviruses is involved in viral attachment and viral fusion to host cells during infection, and studies have suggested that natural selection within the spike protein gene is a mechanism BCoV uses to continually adapt to host immune responses to infection (7–9).

The BCoV has a large genome (27–30 kb), and infection with BCoV can result in both respiratory and enteric infections in cattle as well as in wild ruminants. Symptoms of BCoV infection can vary based on age of the infected cattle but commonly include severe diarrhea and respiratory distress (i.e., coughing, nasal discharge). Prevalence of BCoV in both feedlot and dairy cattle is considered widespread, with estimates ranging from 0 to 8.2% in healthy cattle (10–13) and up to 79% in cattle presenting with disease symptoms (12–14). The economic losses associated with BCoV and BRD infection can be quite substantial (15). In feedlot cattle, BCoV infection is associated with decreased weight gain (16, 17). In the dairy industry, neonatal calf diarrhea contributes up to 50% of the mortality seen in preweaned dairy calves, and respiratory illness accounts for an estimated 24% of pre-weaned heifer death and 58.9% of weaned heifer death in the US (18). The exact proportion of these deaths caused by BCoV infection is unknown. However, previous studies worldwide have indicated that the prevalence of BCoV in calves presenting with diarrhea ranged anywhere from 2.8 to 37% (12, 19–21).

Given the large impact and widespread nature of BCoV infection, identifying loci and genes associated with susceptibility to infection could allow for improved selection of cattle. If loci in strong association with BCoV are identified, they could potentially be included on commercially available assays used by producers for genomics selection to allow for the selection of less susceptible cattle. Previous studies have found multiple loci associated with BRD infection in both dairy (12) and feedlot cattle (13). Many of these studies investigated the prevalence of the various viral and bacterial pathogens associated with the BRD complex, but few genome-wide association analyses (GWAA) investigated loci associated with a single pathogen. The goal of the current study is to investigate genomic regions associated with BCoV infection within three cattle groups: a preweaned Holstein calf population, a beef feedlot cattle population, and the combined population of the dairy calves and beef feedlot cattle. The hypothesis of the study was that susceptibility to BCoV infection is associated with loci in feedlot and dairy cattle with and without BRD clinical symptoms. This study was undertaken to better understand the etiology of the disease and for identification of loci for consideration for genomic selection.

## Materials and Methods

### Study Populations

The dairy calves studied consisted of calves collected from dairies in California (CA; *n* = 1,203 male and 735 female calves) and New Mexico (NM; all female calves, *n* = 647). The preweaned Holstein calves were originally enrolled in a BRD case–control study from July 2011 to January 2012 (12). Only calves with positive or negative BCoV test results were included in the current study.

The feedlot cattle consisted of cattle from Colorado (CO; *n* = 915 steers) and Washington (WA; *n* = 934 heifers). The feedlot population consisted of beef cattle originally enrolled in a BRD case–control study from August 2012 to January 2015 (13, 22). As in the dairy population, only cattle with BCoV test results were included for analysis. Cattle enrolled in the study from the CO feedlot consisted of the following breeds: 837 Angus, 16 Charolais, 20 Hereford, and 42 Red Angus. The cattle enrolled in the study from the WA feedlot consisted of 384 Angus, 96 Charolais, 377 crossbreds, 40 Hereford, and 37 Red Angus.

Cattle in this study were not experimentally challenged with BRD pathogens but were naturally infected. Without performing a pathogen challenge study, it was not possible to be 100% certain that all cattle were exposed to a pathogen. The study's sampling scheme was created in order to maximize the likelihood that case and control calves experienced the same pathogen exposure/environmental stressors. Cattle were enrolled in the original BRD studies based on the McGuirk Health Scoring system [(23); Supplementary Figure 1]. Briefly, the McGuirk Health Scoring system evaluates cattle for BRD clinical signs including temperature, cough, nasal discharge, eye discharge, and ear tilt. Cattle are then assigned a numerical score based on the severity (or lack thereof) of each symptom. Scores range from 0 (no signs of disease) to 12 (multiple severe signs of disease). Cattle that score ≤ 4 were categorized as a control, and cattle with scores ≥ 5 were categorized as a BRD case. In the dairy populations, as previously described in Neibergs et al. (12), calves were observed daily in their hutches. When a sick calf (clinical score ≥ 5) was enrolled in the study, researchers scored the calves in adjacent hutches to determine if they met the criteria as a control ( ≤ 4). In an instance where the adjacent calves were also sick, the researcher would continue to look at the next adjacent calf(s) until a control calf was identified. In the beef population, feedlot cattle were observed daily by a pen rider. Whenever a sick animal was pulled out of the home pen and enrolled in a study, the pen rider would also pull a healthy animal from the same pen (thus exposed to the same environment as the sick/case animal) to enroll as a control. This sampling method was implemented in order to increase the exposure of the control animals to BRD pathogens that were infecting the cases and to ensure that case/control cattle had as similar as environments as possible.

### Sample Collection

Samples collected from all cattle included blood for DNA extraction, and mid-nasal and deep pharyngeal swabs collected for viral and bacterial pathogen identification. In addition to the samples collected, all cattle were evaluated for clinical signs of BRD using the McGuirk Health Scoring system (23; Supplementary Figure 1). All samples were collected upon enrollment into the original BRD study. This sampling process was described in detail previously (12). Briefly, mid-nasal samples were collected from the nasopharyngeal region using a 6-in. sterile unguarded polyester swab (Puritan Medical Products, Guilford, ME, USA) which was inserted 5 in. into the nostril and rotated against the nasal surface for 15 s. After sampling, the end of the swab was removed and placed in 3 ml of viral transport media (Amphotericin B −250 μg/ml, gentamicin −50 mg/ml, HEPES −1 M, HCO_3_, and minimum essential media). Deep pharyngeal samples were collected using two 27-inch sterile guarded swabs with polyester tips (Kalajian Industries, Signal Hill, CA, USA). The distance from the nostril to the medial canthus of the eye of each animal was measured and marked on the swabs prior to collection. Then, the swab was inserted into the nostril into the pharyngeal recess until the mark on the swab reached the nostril. The swab was rotated against the pharyngeal recess surface for 15 s before being removed. After collection, the tip of one swab was removed and placed into the same 3-ml viral transport media tube as the mid-nasal swab. The end of the second deep pharyngeal swab was placed into 1 ml of bacterial transport media (Brucella broth, 15% glycerol). Samples were shipped overnight on ice and then underwent diagnostic testing. Bacteriology sample testing began the same day samples were received, while virology samples were stored at −80°C. Virology samples were tested at the diagnostic lab when several hundred were available to be tested at once. Mid-nasal and deep pharyngeal swabs were tested for the presence of the multiple bacterial and viral pathogens common to BRD infection. These pathogens included *Trueperella pyogenes, Histophilus somni, Mannheimia haemolytica, Pasteurella multocida*, BCoV, bovine respiratory syncytial virus, bovine viral diarrhea virus, bovine herpes virus, and various *Mycoplasma* species. The bacteriology samples were tested for the presence of pathogens using aerobic bacterial and mycoplasma culturing while virology samples were tested using quantitative PCR. For in-depth information on testing techniques, please refer to additional file 1 from Neibergs et al. (12) where these methods have been described previously. For the current study, only BCoV results were utilized.

Genotyping for each animal was performed from DNA extracted from ~3 ml of whole blood collected *via* jugular venipuncture into an EDTA tube (CovidienMonoject, Dublin, Ireland). DNA was isolated using the Puregene DNA extraction kit according to manufacturer's guidelines (Qiagen, Germantown, MD, USA). The DNA was quantified using a NanoDrop 100 spectrophotometer (Wilmington, DE, USA) before genotyping. Genotypes were obtained using the Illumina BovineHD BeadChip (Neogen, Lincoln, NE). The BovineHD BeadChip contained 777,962 SNPs, with an average spacing of 3 kb across the genome. These SNPs were mapped using the ARS-UCD 1.2 assembly (https://www.animalgenome.org/repository/cattle/UMC_bovine_coordinates/).

### Quality Control

Prior to conducting GWAA, a series of quality control filtering steps were applied to the genotypes and the cattle. First, genotypes were filtered by call rate (<90%) which removed 165 cattle. Duplicated genotyped animals were identified, and five duplicated cattle were removed from the analysis. SNPs were removed if the genotyping call rate was <90% (19,983 SNPs removed), if the minor allele frequency was <1% (94,774 SNPs removed), or if they deviated from the Hardy–Weinberg equilibrium (*p* < 1 × 10^−75^; 20,212 SNPs removed). Twelve cattle were removed due to discrepancies between genetic and anatomical designations of sex. Finally, 24 cattle were removed for phenotypic inconsistencies or a lack of phenotypic information such as missing diagnostic information or animals misclassified as case or control. After quality control, 4,231 cattle (CA = 1,876; NM = 610; CO = 866; WA = 879) and 642,993 SNPs remained for analysis.

### Phenotypes

Two comparisons associated with BCoV were investigated: (1) cattle positive for BCoV (BCoV^+^) were compared to cattle negative for BCoV (BCoV^−^) and (2) cattle positive for BCoV and affected with BRD (BCoV^+^BRD^+^) were compared to cattle negative for BCoV and BRD (BCoV^−^BRD^−^). For the BCoV^+^ phenotype, the dairy population consisted of 419 BCoV^+^ calves (262 from NM and 157 from CA) and 2,067 BCoV^−^ calves (348 from NM and 1,719 from CA), and the feedlot population consisted of 236 BCoV^+^ cattle (128 from CO and 108 from WA) and 1,509 BCoV^−^ cattle (738 from CO and 771 from WA). Analyses on a combined dairy and feedlot population analysis were also performed and contained 655 BCoV^+^ and 3576 BCoV^−^. For the BCoV^+^BRD^+^ phenotype, the dairy population had 242 BCoV^+^BRD^+^ (88 from CA and 154 from NM) and 1,074 BCoV^−^BRD^−^ (876 from CA and 198 from NM), the feedlot population had BCoV^+^BRD^+^ (82 from CO and 59 from WA) and 790 BCoV^−^BRD^−^ (397 from CO and 392 from WA), and the combined population had 383 BCoV^+^BRD^+^ and 1,864 BCoV^−^BRD^−^.

The dairy and feedlot populations were investigated for loci associated with BRD as previously described (12, 13, 22); however, they were not investigated for susceptibility to BCoV specifically. For the combined dairy and feedlot analysis, the same covariates were assessed as in the individual beef and dairy cattle populations.

### Statistical Analysis

A Student's *T*-test (*p* < 0.05) was used to test for effects of location and sex on the presence of BCoV prior to the GWAA. If sex or location was significant, it was subsequently used as a covariate for the GWAA. The GWAA for the three populations (dairy, feedlot, and combined) were performed using the SNP & Variation Suite version 8 from Golden Helix Inc^1^. Statistical analyses were conducted using the efficient mixed-model association expedited (EMMAX) method (25) and three genotypic models (additive, dominant, and recessive). The general mixed model was described by **y** = **Xβ** + **Z***u* + ϵ, where y is the n × 1 vector of observed phenotypes, X was the n × f matrix of fixed effects (f), β was a f × 1 vector containing the fixed effect coefficients, Z was a n × t matrix relating the random effects (t) to the phenotype, and *u* was the random effect of the mixed model. The model assumes residuals to be independent with an identical distribution such that $Var\left(u\right)=\;{\sigma_{g}}^{2}\text{K}$ and $\left(\epsilon \right)=\;{\sigma_{e}}^{2}\text{I}$, and $Var\left(y\right)=\;{\sigma_{g}}^{2}\text{Z}\text{K}{\text{Z}}^{\prime }+\;{\sigma_{e}}^{2}\text{I}$ (26). For this study, K was a matrix of pairwise genomic relationships and Z was the identity matrix, I (26). Given that the model of inheritance for BCoV infection and BRD is unknown, all three genotypic models were analyzed. Heritability was estimated using a genomic-best linear unbiased prediction (GBLUP) model (27, 28) and an average information, restricted maximum likelihood (AI-REML) algorithm (29, 30). More detailed information about the SVS methods for EMMAX and AI-REML/GBLUP can be found at http://doc.goldenhelix.com/SVS/latest/svsmanual/mixedModelMethods/overview.html.

The Wellcome Trust Consortium (24) significance threshold for uncorrected *p*-values was used to determine if SNP were strongly (*p* < 5 × 10^−7^) or moderately (*p* between 1 × 10^−5^ and 5 × 10^−7^) associated with infection. The false discovery rate (FDR) was also calculated for all *p*-values within SVS. FDR used the following model: FDR = Σ (V/R | R > 0) Pr (R > 0), where R is the number of rejected hypotheses and V is the number of reject hypotheses that are truly false positive (type I error). Positional candidate genes were identified within a 36-kb region (18 kb 3′ and 5′ of the SNP) in dairy cattle or 24 kb (12 kb 3′ and 5′ of the SNP) in feedlot cattle around significant SNP. In the combined population, positional candidate genes were identified within a 30-kb region surrounding the significant SNP. This region was based on the average haplotype block size of the dairy and feedlot populations in this study and was determined following the method proposed by Gabriel et al. (31).

## Results

For the dairy calf population, the average age of BCoV positive (BCoV^+^) calves (49.3 days) and BCoV negative (BCoV^−^) calves (48.5 days) did not differ (*p* = 0.14). As only the CA calves had both male and female calves, the effect of sex was tested based on BCoV status (*p* = 0.05) and on BCoV and BRD (BCoV^+^BRD^+^) status (*p* = 0.04). When the CA calves were combined with the NM calves, sex remained significant with BCoV (*p* = 2 × 10^−30^) and in BCVoV^+^BRD^+^ (*p* = 7 × 10^−22^). Dairy location also played a role in the infection of BCoV (*p* = 2 × 10^−76^) as more calves were BCoV^+^ in NM (63%) than in CA (37%). Therefore, for the dairy population analyses, location and sex were included as covariates. In the feedlot populations, there was no significant influence of breed (*p* = 0.09), sex (*p* = 0.12), or location (*p* = 0.12) on BCoV status so these potential covariates were excluded from the beef analyses. In the combined beef and dairy population, there was no significant influence of breed (*p* = 0.20) on BCoV status; however, sex (*p* = 1.6 × 10^−10^) and location (*p* = 2.6 × 10^−41^) did influence the phenotypes. When location was used as a covariate in the combined population, sex was no longer a significant factor in BCoV or BRD status. Therefore, location alone was used as a covariate in the analysis of the combined population.

*Phenotype 1: bovine coronavirus cases* (BCoV^+^) *vs. controls* (BCoV^−^).

Heritability estimates for BCoV infection status varied by population. The heritability estimate was 0.11 ± 0.06 in the feedlot population and 0.01 ± 0.02 in the dairy population. As the heritability estimate with the standard deviation encompassed zero for the dairy population, one must consider the heritability estimate for dairy as zero. As would be expected, the heritability estimate for the combined feedlot and dairy population was intermediate (0.03 ± 0.02) to the two individual feedlot and dairy populations.

**Figure 1:**
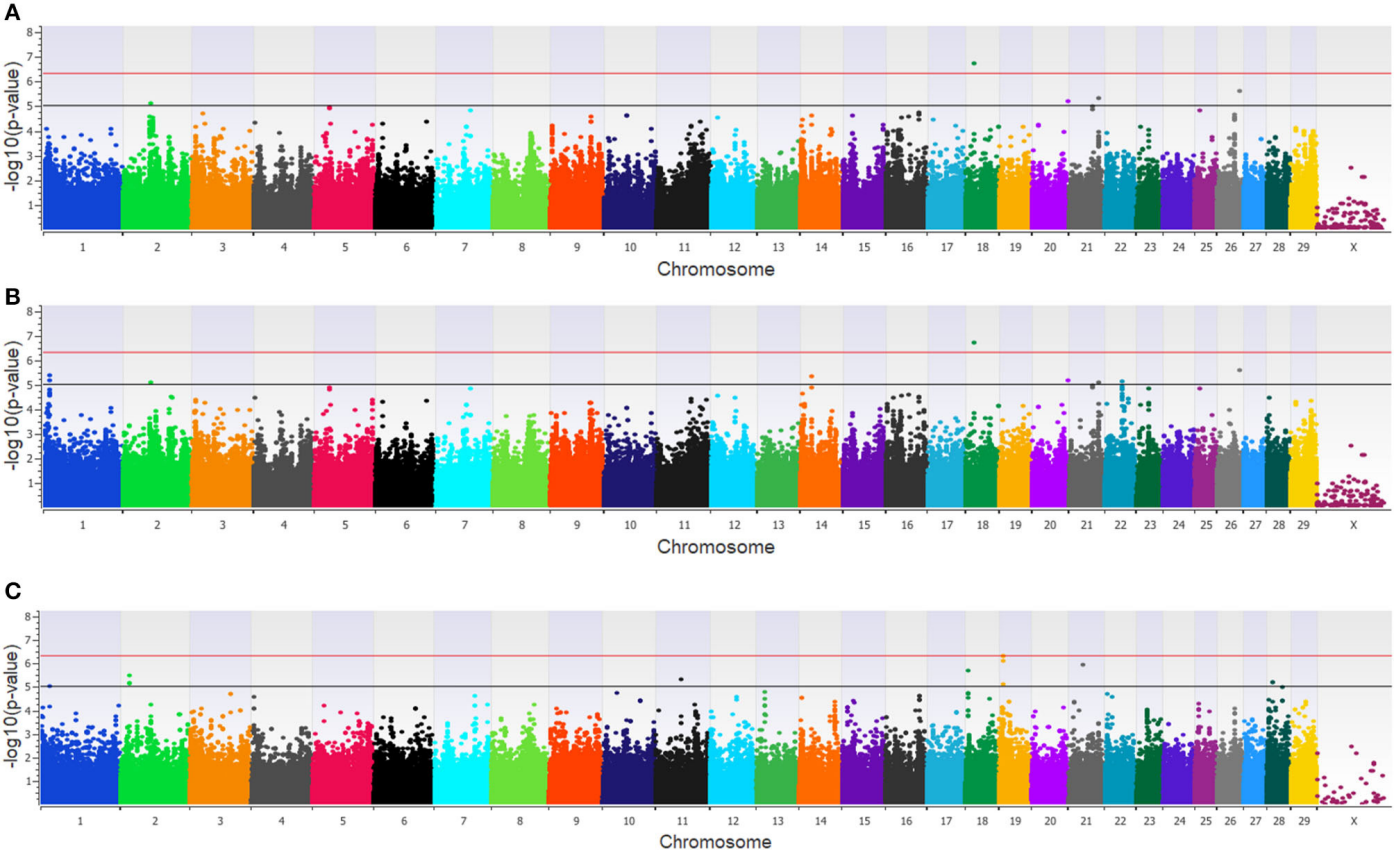
Manhattan plots identifying loci associated with bovine coronavirus infection in additive **(A)**, dominant **(B)**, and recessive **(C)** EMMAX models for the dairy population. Single-nucleotide polymorphisms (SNP) are represented by a single dot. Bovine chromosomes are listed on the x-axis. SNPs located between the black and red line provide evidence of moderate association (*p* between 1 × 10^−5^ and 5 × 10^−7^), and SNPs above the red line provide evidence of strong association (*p* < 5 × 10^−7^) based on the Wellcome Trust Case Control Consortium (24) guidelines.

Twenty-three unique SNPs (16 loci) were associated (*p* < 1 × 10^−5^) with BCoV^+^ in the additive, dominant, and recessive models for the dairy population (Supplementary Table 1). The additive model identified four moderately (1 × 10^−5^ < p > 5 × 10^−7^) and one strongly (*p* < 5 × 10^−7^) associated SNP (five loci; Figure 1A), while the dominant model identified eight moderately and one strongly associated SNP (five loci; Figure 1B). The recessive model identified 14 moderately associated SNPs (eight loci; Figure 1C). All of the SNPs associated with BCoV^+^ in the additive model were also associated with BCoV^+^ in the dominant model. Eleven positional candidate genes were identified in the dairy population, with significant SNPs located within eight of the genes and the remaining three genes were located within the haplotype block region of the significant SNPs such as *MSI2* (Supplementary Table 1).

**Figure 2:**
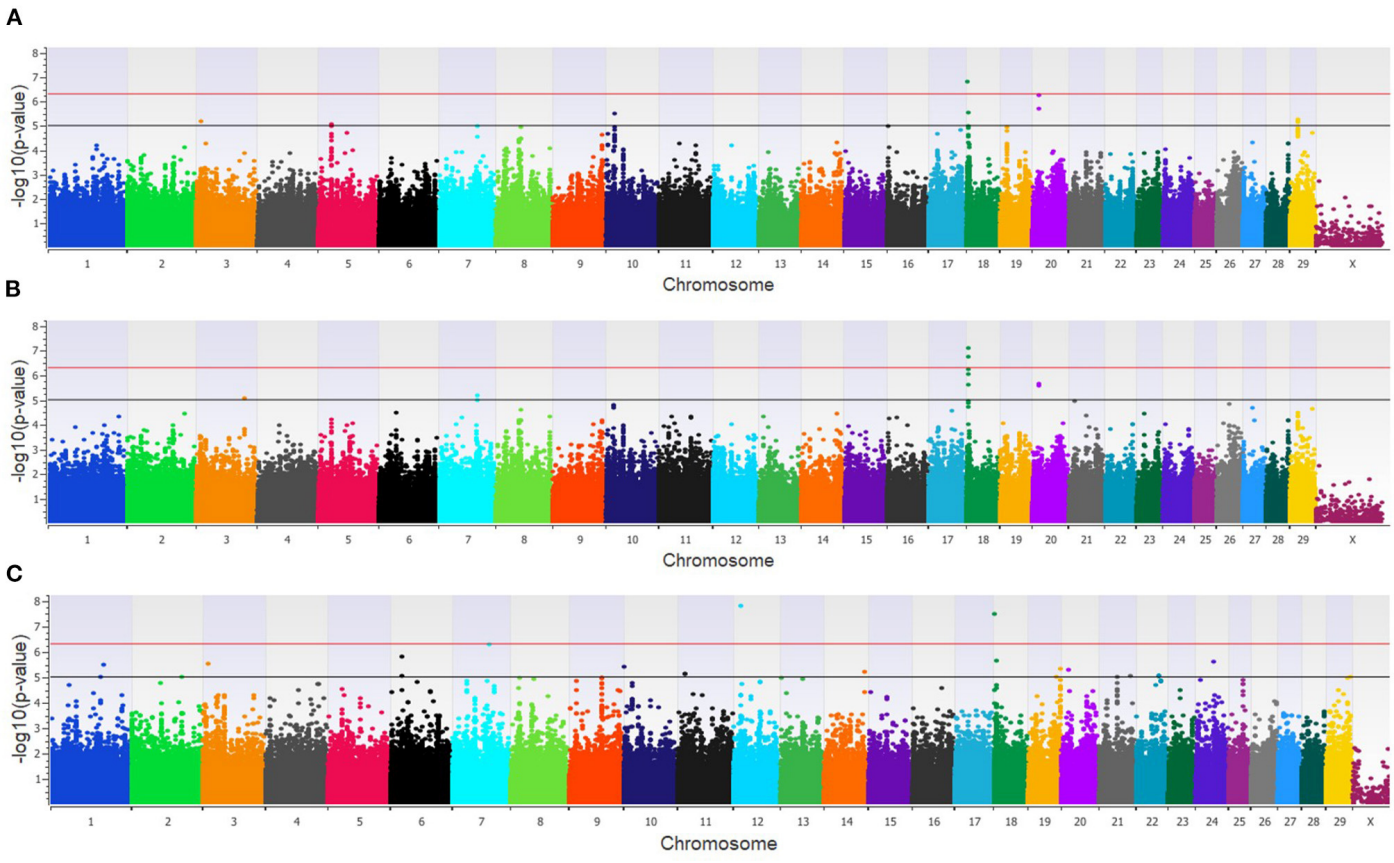
Manhattan plots identifying loci associated with bovine coronavirus infection in additive **(A)**, dominant **(B)**, and recessive **(C)** EMMAX models for the feedlot population. Single-nucleotide polymorphisms (SNP) are represented by a single dot. Bovine chromosomes are listed on the x-axis. SNPs located between the black and red lines provide evidence of moderate association (*p* between 1 × 10^−5^ and 5 × 10^−7^), and SNPs above the red line provide evidence of strong association (*p* < 5 × 10^−7^) based on the Wellcome Trust Case Control Consortium (24) guidelines.

The GWAA for the feedlot population identified 39 unique SNPs (24 loci) associated with BCoV^+^ (Supplementary Table 1). There were nine moderately and one strongly associated SNP (seven loci) in the additive model (Figure 2A), while the dominant model identified seven moderately and two strongly associated SNPs (four loci) associated with BCoV^+^ (Figure 2B). The recessive model in the feedlot cattle, as with the dairy cattle, had more associations with BCoV^+^ than in the dominant and additive models. There were 23 moderately and 2 strongly associated SNPs (17 loci) in the recessive model (Figure 2C). Five SNPs were shared across multiple models, as three SNPs were common to the additive and dominant models, and two SNPs were common to the additive and recessive models. A total of 18 positional candidate genes were identified in the feedlot population, with the significant SNP located within 13 of the positional candidate genes including *PRKCA* and *WWOX* (Supplementary Table 1).

**Figure 3:**
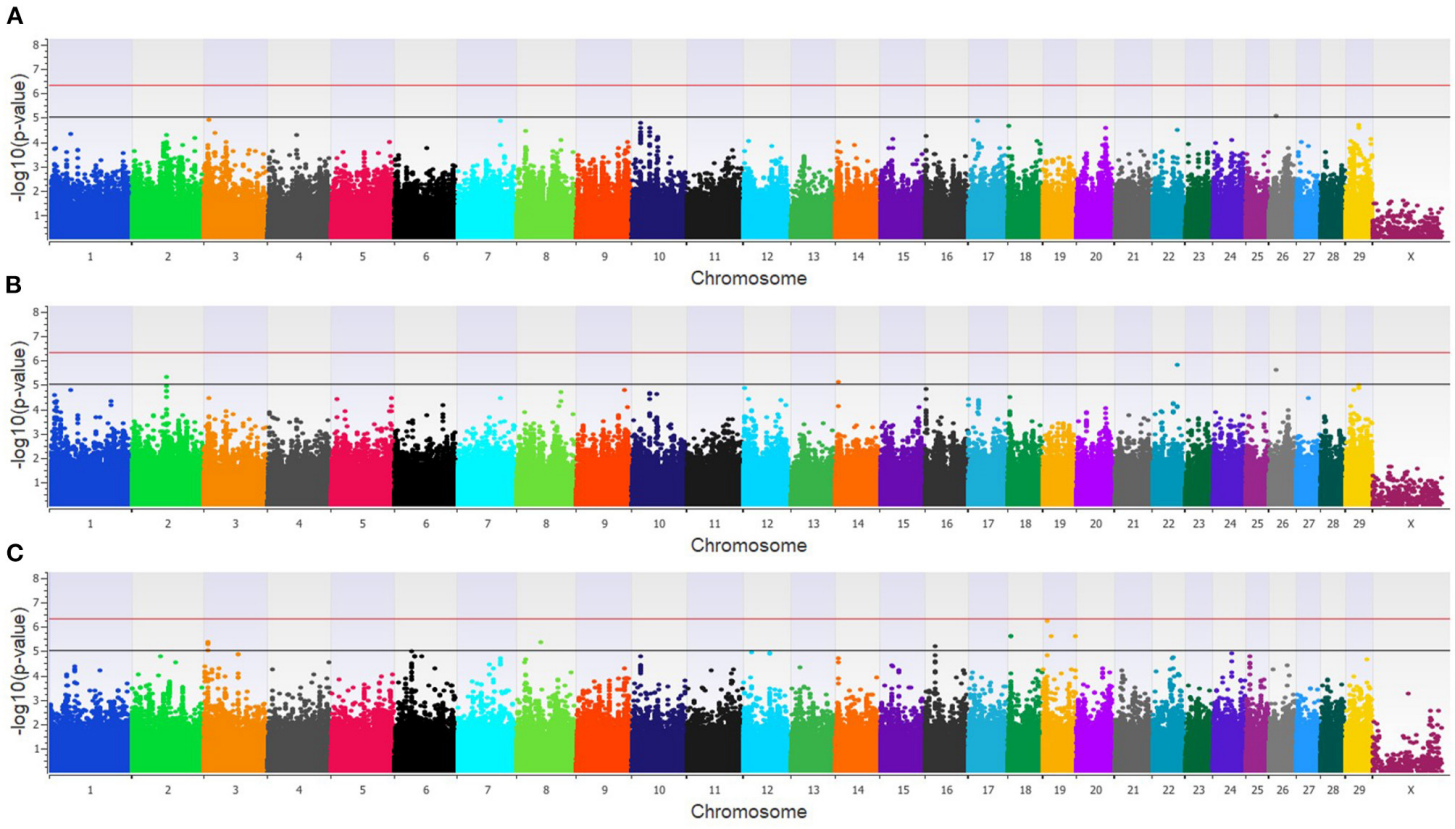
Manhattan plots identifying loci associated with bovine coronavirus infection in additive **(A)**, dominant **(B)**, and recessive **(C)** EMMAX models for the combined dairy and feedlot population. Single-nucleotide polymorphisms (SNP) are represented by a single dot. Bovine chromosomes are listed on the x-axis. SNPs located between the black and red lines provide evidence of a moderate association (*p* between 1 × 10^−5^ and 5 × 10^−07^), and SNPs above the red line provide evidence of a strong association (*p* < 5 × 10^−7^) based on the Wellcome Trust Case Control Consortium (24) guidelines.

The combined dairy and feedlot GWAA identified 15 unique SNPs (11 loci) associated with BCoV^+^ (Supplementary Table 1). A single SNP was moderately associated in the additive model (Figure 3A), and this SNP was also moderately associated with BCoV^+^ in the dominant model. The dominant model further identified four moderately associated SNPs (three loci) (Figure 3B), and the recessive model identified 11 moderately associated SNPs (seven loci) with BCoV^+^ (Figure 3C). Eight positional candidate genes, including *MSI2, PRKCA*, and *WWOXX* which harbored the SNP associated with BCoV^+^, were identified in the combined population (Supplementary Table 1).

Three loci were identified in more than one of the feedlot, dairy, and combined cattle populations in the recessive model (Table 1), and each of these loci were defined by one or more SNPs that were located within a positional candidate gene *(MSI2, PRKCA*, and *WWOX*). No locus was found to be associated with BCoV^+^ across all three populations.

**Table 1:** Loci associated with bovine coronavirus infection across populations.

**BTA^a^**	**Locus range (Bb)**	**Lead SNP ID^b^**	**# SNP in locus**	**Population(s)^c^**	**Model(s)**	**Positional candidate gene(s)^d^**
18	5,420,736–5,749,984	*rs109736099*	3	C & F	Recessive	***WWOX***
19	8,294,507–8,360,868	*rs134736482*	3	C & D	Recessive	***MSI2***
19	62,757,064–62,763,041	*rs41926279*	2	C & F	Recessive	***PRKCA***

a *Single-nucleotide polymorphism (SNP) location as measured by numbered nucleotides in reference to the ARS 1.2 genome assembly (https://www.animalgenome.org/repository/cattle/UMC_bovine_coordinates/; accessed 21, January 2020)*.

b *Most significant SNP in each locus is identified by rs number which is a reference number assigned to markers submitted to the National Center for Biotechnology Information SNP database*.

c *Populations abbreviated as follows: C, combined feedlot and dairy; F, feedlot; D, dairy*.

d *Positional candidate genes are defined as genes that are located within the average haplotype block range (combined = 15 kb; feedlot = 12 kb; dairy = 18 kb) on either side of the associated SNP(s) or had the significant SNP located within the gene itself (bolded)*.

*Phenotype 2: bovine coronavirus and bovine respiratory disease cases* (BCoV^+^BRD^+^) *vs. bovine coronavirus and bovine respiratory disease controls* (BCoV^−^BRD^−^).

Heritability estimates for BCoV^+^BRD^+^ was highest in the feedlot populations at 0.44 ± 0.13. The heritability estimate for BCoV^+^BRD^+^ was (0.005 ± 0.04) for the dairy, which included zero when the standard error was considered, similar to the BCoV^+^ heritability estimate. The combined population's heritability estimate was intermediate to the feedlot and dairy heritability estimate for BCoV^+^BRD^+^ at 0.07 ± 0.04.

**Figure 4:**
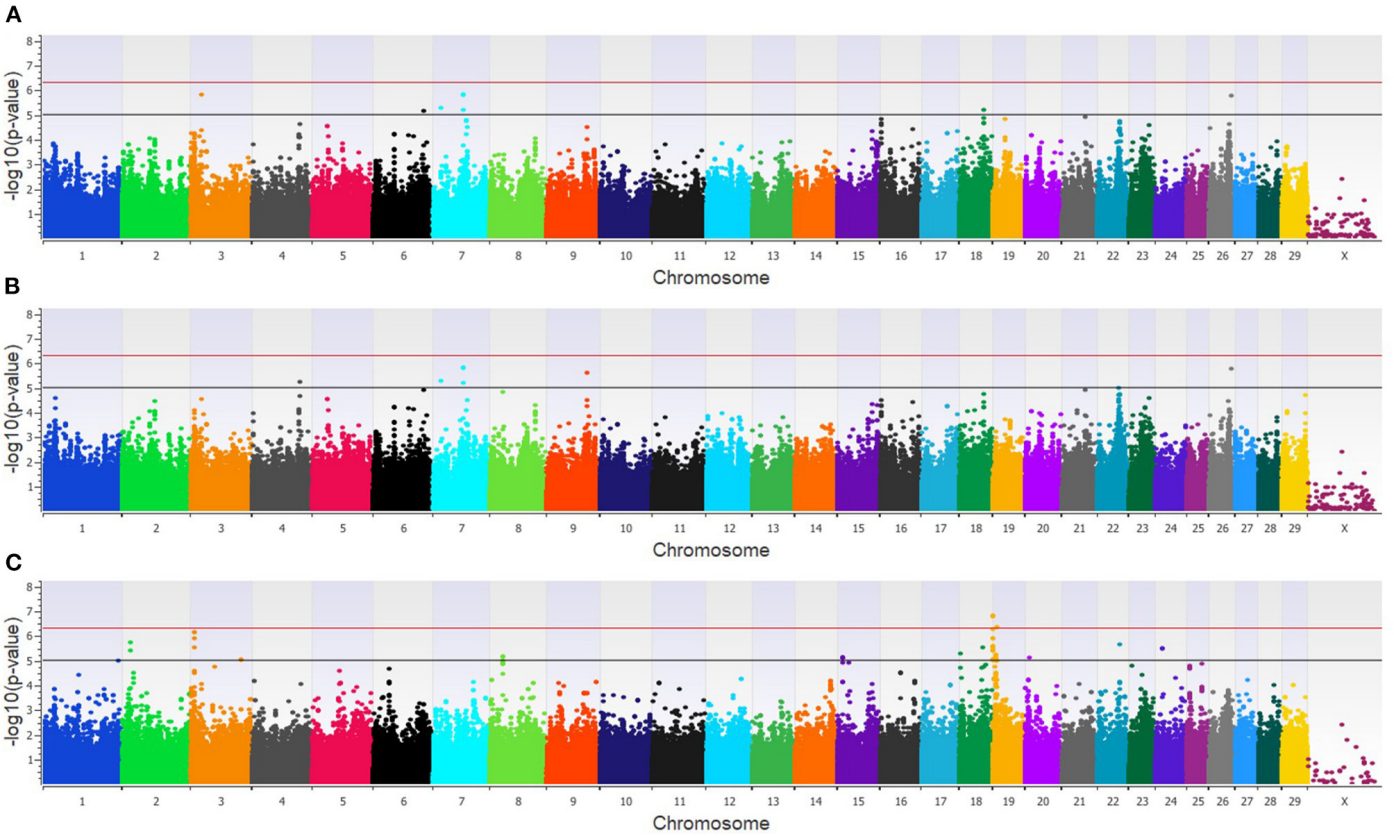
Manhattan plots identifying loci associated with bovine coronavirus infection and bovine respiratory disease in additive **(A)**, dominant **(B)**, and recessive **(C)** EMMAX models for the dairy population. Single-nucleotide polymorphisms (SNP) are represented by a single dot. Bovine chromosomes are listed on the x-axis. SNPs located between the black and red lines provide evidence of moderate association (*p* between 1 × 10^−5^ and 5 × 10^−7^), and SNPs above the red line provide evidence of a strong association (*p* < 5 × 10^−7^) based on the Wellcome Trust Case Control Consortium (24) guidelines.

The GWAA for the dairy population identified 83 unique SNPs (25 loci) associated with BCoV^+^BRD^+^ (Supplementary Table 2). The additive model identified nine SNPs (six loci) moderately associated with BCoV^+^BRD^+^ (Figure 4A), while the dominant model identified eight SNPs (five loci) moderately associated with BCoV^+^BRD^+^ (Figure 4B). The recessive model identified 60 moderately and 12 strongly associated SNPs with BCoV^+^BRD^+^ (15 loci; Figure 4C). There were six shared SNPs associated with BCoV^+^BRD^+^ in the additive and dominant models. No SNP was shared between the recessive and additive models. Within the dairy population, 31 positional candidate genes were identified (Supplementary Table 2). Of these, 17 genes contained the significant SNP within the gene while the remaining 14 positional candidate genes were located in the region surrounding the significant SNP including *CA10, CWC22, LOC107131482, LOC789077, LOC524702*, and *NOS1AP*.

**Figure 5:**
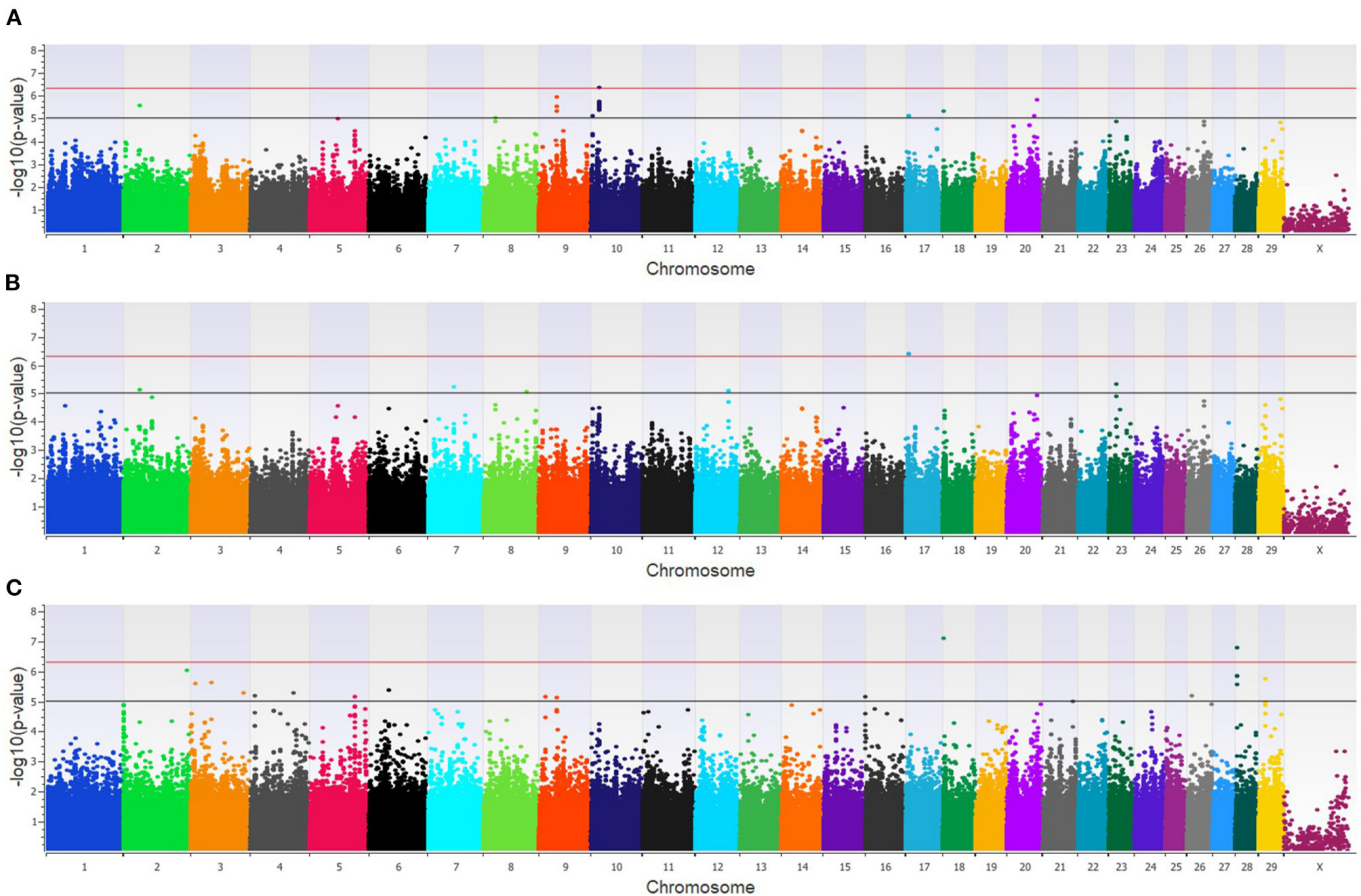
Manhattan plots identifying loci associated with bovine coronavirus infection and bovine respiratory disease in additive **(A)**, dominant **(B)**, and recessive **(C)** EMMAX models for the feedlot population. Single-nucleotide polymorphisms (SNP) are represented by a single dot. Bovine chromosomes are listed on the x-axis. SNPs located between the black and red lines provide evidence of moderate association (*p* between 1 × 10^−5^ and 5 × 10^−7^), and SNPs above the red line provide evidence of strong association (*p* < 5 × 10^−7^) based on the Wellcome Trust Case Control Consortium (24) guidelines.

In the feedlot population, a total of 44 unique SNP (26 loci) were identified by the three GWAA models (Supplementary Table 2). The additive GWAA identified 23 significant SNP (eight loci; Figure 5A), including one that was strongly associated, while the dominant model identified seven significant SNPs including two that were strongly associated (six loci; Figure 5B). The recessive model identified 19 associated SNP including two in strong association (16 loci; Figure 5C). Between models, three SNPs were associated with BCOV^+^BRD^+^ in both the additive and dominant models while two SNPs were associated in the recessive and additive models. Eighteen positional candidate genes were identified in the feedlot population (Supplementary Table 2). Thirteen of the 18 genes harbored the significant SNP within the gene, while five genes were located in the regions near associated SNP such as *AKAP9, DISC1, LOC100849043, LOC104969525, PRSS48*, and *SH3D19*.

**Figure 6:**
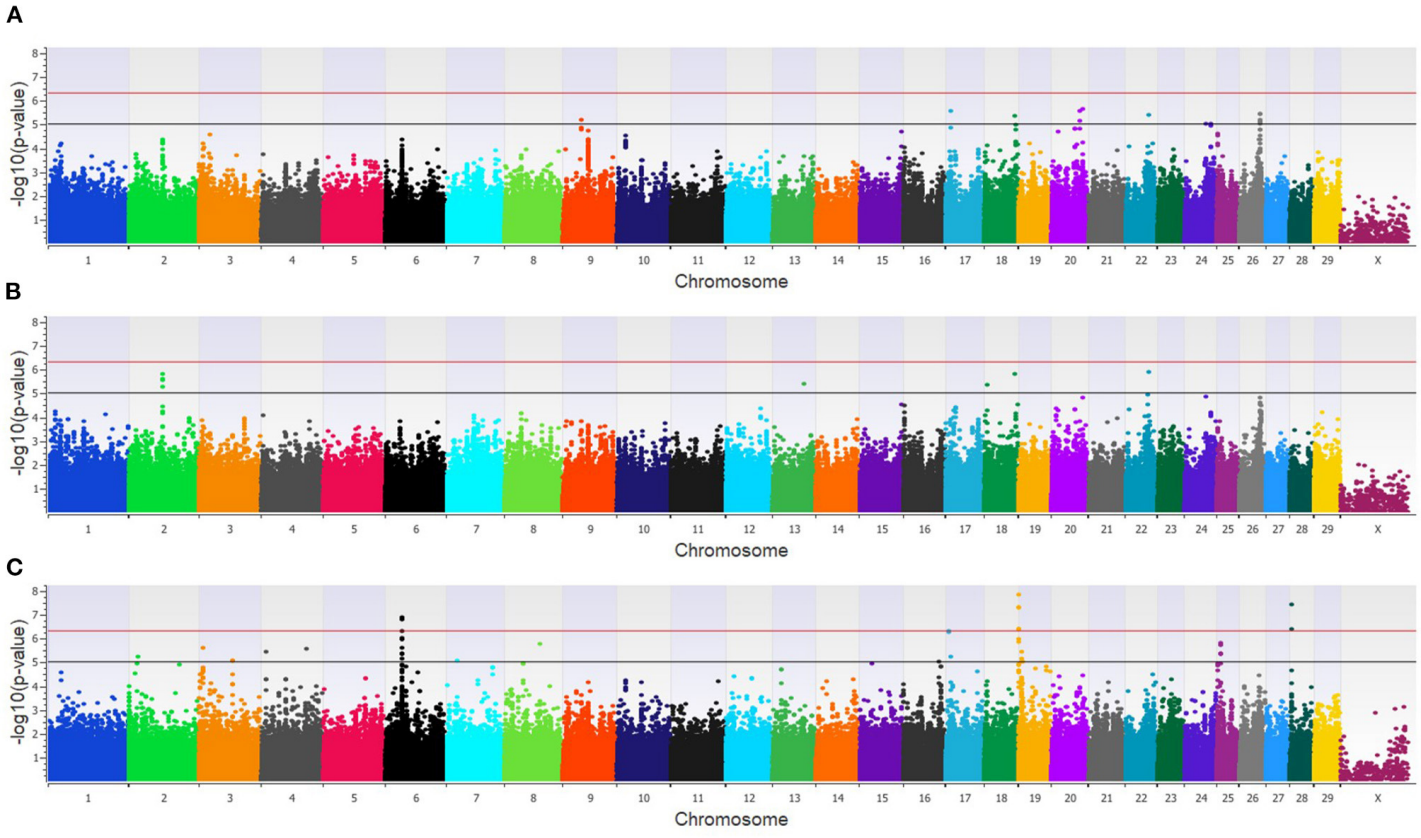
Manhattan plots identifying loci associated with bovine coronavirus infection and bovine respiratory disease in additive **(A)**, dominant **(B)**, and recessive **(C)** EMMAX models for the combined dairy and feedlot population. Single-nucleotide polymorphisms (SNP) are represented by a single dot. Bovine chromosomes are listed on the x-axis. SNPs located between the black and red lines provide evidence of a moderate association (*p* between 1 × 10^−5^ and 5 × 10^−7^), and SNPs above the red line provide evidence of a strong association (*p* < 5 × 10^−7^) based on the Wellcome Trust Case Control Consortium (24) guidelines.

Within the combined population, a total of 81 unique SNPs (29 loci) were associated with BCoV^+^BRD^+^ in the three GWAA models (Supplementary Table 2). The additive model identified 15 SNPs (nine loci) that were in moderate association with BCoV^+^BRD^+^ (Figure 6A), while the dominant model identified nine SNPs (five loci) that were moderately associated with BCoV^+^BRD^+^ (Figure 6B). As in the BCoV^+^ phenotype, the recessive model identified the greatest number of SNPs associated with BCoV^+^BRD^+^. There were 45 moderately and 15 strongly associated SNPs (18 loci) with BCoV^+^BRD^+^ (Figure 6C). Three associated SNPs were shared between models, two between the additive and dominant models, and one between the additive and recessive models. A total of 30 positional candidate genes were found in the combined population (Supplementary Table 2). Fourteen of the positional candidate genes were identified by having an associated SNP within their haplotype block, whereas 16 positional candidate genes harbored SNP associated with BCoV^+^BRD^+^ within the gene including *AKAP9, CA10, CWC22, DISC1, LOC104969525, LOC107131482, LOC789077, LOC524702, LOC100849043, MSI2, NOS1AP, PRSS48*, and *SH3D19*.

Across the three populations, 14 loci were associated with BCoV^+^BRD^+^ in more than one of the populations with 13 harboring positional candidate genes (Table 2). These loci were associated with BCoV^+^BRD^+^ in additive and dominant or additive and recessive models and contained between one and 18 SNPs. No loci were associated with BCoV^+^BRD^+^ in all three populations.

**Table 2:** Loci associated with bovine coronavirus and bovine respiratory disease infections across populations.

**BTA^a^**	**Locus range (Bb)**	**Lead SNP ID^b^**	**# SNP in locus**	**Population(s)^c^**	**Model(s)**	**Positional candidate gene(s)^d^**
2	16,887,183–16,892,466	*rs136233359*	2	C & D	Recessive	*CWC22*
3	7,228,232–7,236,300	*rs109804510*	4	C & D	Recessive	***NOS1AP***
4	9,439,592	*rs41587962*	1	C & F	Recessive	***AKAP9***
4	88,516,050	*rs42043233*	1	C & F	Recessive	–
9	35,944,485–35,956,425	*rs110498854*	3	C & F	Additive & recessive	*LOC104969525*
17	6,515,432–6,516,072	*rs135350640*	2	C & F	Additive, dominant, & recessive	***SH3D19**, PRSS48*
19	154,015–317,404	*rs135692084*	6	C & D	Recessive	***LOC107131482**, LOC789077, LOC524702*
19	340,070–468,681	*rs110355574*	28	C & D	Recessive	-
19	853,741–868,633	*rs134561123*	2	C & D	Recessive	***CA10***
19	6,846,738–6,855,449	*rs110565142*	3	C & D	Recessive	-
19	8,294,507–8,360,868	*rs133988665*	3	C & D	Recessive	***MSI2***
20	56,000,163–56,003,536	*rs137256606*	2	C & F	Additive	***LOC100849043***
20	60,955,121	*rs133738096*	1	C & F	Additive	-
28	4,224,198–4,534,637	*rs110583329*	4	C & F	Recessive	***DISC1***

a *Single nucleotide polymorphism (SNP) location as measured by numbered nucleotides in reference to the ARS 1.2 genome assembly (https://www.animalgenome.org/repository/cattle/UMC_bovine_coordinates/; accessed 21, January 2020)*.

b *Most significant SNP in each locus is identified by rs number which is a reference number assigned to markers submitted to the National Center for Biotechnology Information SNP database*.

c *Populations abbreviated as follows: C, combined feedlot and dairy; F, feedlot; D, dairy*.

d *Positional candidate genes are defined as genes that are located within the average haplotype block range (combined = 15 kb; feedlot = 12 kb; dairy = 18 kb) on either side of the associated SNP(s) or had the significant SNP located within the gene itself (bolded)*.

When loci associated with BCoV^+^ and BCoV^+^BRD^+^ were compared, a single region on BTA19 ranging from 8,294,507 to 8,360,868 was identified with both phenotypes. Associations for both phenotypes in this region were identified in the recessive models in the dairy and combined populations. This region also contained a single positional candidate gene, Musashi RNA-binding protein 2 (*MSI2*), which contained an SNP associated with the phenotypes. The positional candidate genes identified across populations for BCoV^+^ (3; Table 1) and for BCoV^+^BRD^+^ (13; Table 2) were further analyzed to determine if there were involved in any similar biological pathways. Two genes, *AKAP9* (BCoV^+^) and *PRKCA* (BCoV^+^BRD^+^), were involved in nine of the same pathways: activation of cAMP-dependent PKA, mitotic cell cycle, DAG and IP3 signaling, developmental biology, dopamine-DARPP32 feedback onto cAMP pathway, innate immune system, RET signaling, signaling by G-coupled protein receptors (GPCR), and transmission of chemical synapses. One of these pathways, signaling by GPCR, also involved a third positional candidate gene WWOX, which was associated with BCoV^+^.

## Discussion

The heritability estimates described in this study measure the additive genetic variance explained for BCoV^+^ and BCoV^+^BRD^+^ infection in cattle. The heritability estimate for the dairy population was low and spanned zero when the standard deviation was included. In dairy, more loci were associated with BCoV^+^ or BCoV^+^BRD^+^ in the non-additive (recessive and dominant) models than in the additive models. This suggests that the additive gene effects were overshadowed by the genetic variance explained by the non-additive (dominant and recessive) loci. This was particularly true in the diary population for the recessive model, as none of the recessive loci were identified in the additive model, and so did not contribute to the heritability estimate. However, in the dominant model, some of the loci associated with BCoV^+^ or BCoV^+^BRD^+^ were also identified in the additive model and did contribute to the heritability estimates. In contrast to the dairy populations, in the feedlot populations the heritability estimate was moderate even when including the range of the standard deviations. Although the feedlot population was similar to the dairy population in that more non-additive loci were associated with BCoV^+^ or BCoV^+^BRD^+^, the proportion of variance explained by the additive loci was slightly greater than in the dairy populations, thereby increasing the heritability estimates. The incidence of BCoV^+^ was disparate between the NM (42%) and CA (8%) dairy calves, whereas the incidence was similar between the CO (15%) and WA (12%) feedlots. The differences between the incidence of BCoV^+^ may reflect environmental and genetic differences in these calves, which can also reduce heritability estimates. Although the heritability estimates for BCoV^+^ were low in dairy cattle, it is possible to make genetic improvement in traits with low heritabilities. Multiple studies have shown that selection for lowly heritable traits is possible and justifiable (32, 33).

Around 100 positional candidate genes were identified within regions associated with BCoV^+^ and/or BCoV^+^BRD^+^. To allow for a more detailed discussion, the discussion will focus on the potential roles of 15 positional candidate genes associated with these phenotypes in multiple populations.

Of the 15 positional candidate genes identified in multiple populations, only one (from BTA19 at 8.3 kb) was associated with BCoV^+^ and BCoV^+^BRD^+^. This locus harbors *MSI2*, an RNA-binding protein associated with cell-cycle regulation. While its counterpart *MSI1* has been widely studied for its roles in translation (34, 35), fewer studies have investigated *MSI2*. *MSI2* regulates hematopoiesis, and dysregulation of *MSI2* can impact cellular proliferation and apoptosis (36). A loss of *MSI2* function negatively impacts a host's innate immune responses in response to infection by affecting hematopoietic cell homeostasis and thus leukocyte development in both humans and mice (37, 38). Overexpression of *MSI2* leads to increased hematopoietic cell expansion and self-renewal and can lead to increased pathogenesis of certain hematopoietic cell diseases (36, 39). Previous studies on human bronchial epithelial cells found that during infection with severe acute respiratory syndrome coronavirus 2 (SARS-CoV-2), the causative coronavirus associated with COVID-19, expression of *MSI2* was downregulated in infected cells suggesting cells were more prone to infection due to issues with the innate immune response to infection (40). That *MSI2* is involved in other coronavirus infections provides support for a possible role for *MSI2* in modulating BCoV infection.

Two additional positional candidate genes, protein kinase C alpha (*PRKCA*) and WW domain-containing oxidoreductase (*WWOX*), were associated with BCoV^+^ in more than one population (Table 1). *PRKCA* is a member of a kinase family that has roles in many cellular processes including cell permeability and cell signaling. In patients with pneumococcal pneumonia, PRKCA has a role in the activation antibiotic-induced release of pneumolysin (41). The presence of pneumolysin in the lungs leads to increased pulmonary endothelial permeability and potentially increases edema in the lungs (41). Similarly, *PRKCA* alters pulmonary endothelial cell permeability in humans and mice in response to lung injury *via* reactive oxygen species, alpha-thrombin, and TNF-alpha (42). Multiple studies have also implicated that *PRKCA* has a role in respiratory syncytial virus (RSV) infection. Upon initial infection with RSV, *PRKCA* is activated and co-localizes with the virus. The binding of PRKCA and RSV is needed to facilitate the fusion of RSV with host cell membranes (43, 44). Both BCoV and bovine RSV are part of the BRD complex of pathogens. Bovine respiratory disease is common to dairy and beef cattle and causes considerable economic loss to the cattle industry. Similar to *MSI2, PRKCA* has also been linked to severe acute respiratory syndrome (SARS) in humans, which is caused by a SARS-CoV-1. Liu et al. (45) found that the SARS-CoV spike protein stimulates *PRKCA* to modulate a NFKB pathway through the influx of calcium ions. Ultimately, the activation of this pathway and other calcium-independent pathways induces inflammation and tissue damage within the lungs leading to symptoms of SARS-CoV (45).

The *WWOX* gene is a tumor suppressor which can interact with a variety of transcription factors in inflammation and cancer. A knockdown study in mice found that decreased expression of WWOX leads to an influx of neutrophils, increased vascular leakage, and increased inflammatory cytokine production (46). Ultimately, these increased immune responses triggered acute respiratory distress syndrome (ARDS) in mice (46). This increase in neutrophils and vascular leakage can negatively impact the host increasing the chances of damage during an infection.

Twelve additional positional candidate genes were identified across populations in BCoV^+^BRD^+^ cattle (Table 2). These genes can be grouped by function such as those that have roles related to olfactory receptors, pulmonary diseases, and viral infections. Three of the positional candidate genes are olfactory receptor like-genes (*LOC10713482, LOC524702*, and *LOC789077*). These genes have no known function relating to BCoV or BRD infection and are not fully characterized in cattle. However, the olfactory epithelium and olfactory neurons have been linked to early immune responses to SARS-CoV2 infection and loss of smell is a common symptom of COVID-19 (47). It is possible that cattle also experience a loss of smell during BCoV or BRD infection, and this could contribute to the decrease in appetite seen in some cattle.

Four additional positional candidate genes, carbonic anhydrase 10 (*CA10*), CWC22 spliceosome-associated protein (*CWC22*), DISC1 scaffold protein (*DISC1*), and nitric oxide synthase 1 adaptor protein (*NOS1AP*), have roles in pulmonary dysfunction, viral infections, or both. A recent study in humans determined that dysregulation of carbonic anhydrases, along with membrane metallo-endopeptidase and angiotensin-converting enzyme 2, during a SARS-CoV2 infection results in increased levels of carbon dioxide in the bloodstream as well as pulmonary edema and eventually heart failure (48). Hypermethylation of *NOS1AP* in patients with chronic obstructive pulmonary disorder results in gene silencing in lung tissues (49). Gene silencing of *NOS1AP* expression could disrupt downstream pathways within the lung related to autophagy and apoptosis (49). Like *CA10, DISC1* has ties to both pulmonary disease and viral infections (50, 51). *CWC22*, a regulator of mRNA splicing and RNA metabolism, does not have a direct link to BCoV or BRD infection. However, a study on how influenza viruses, like H1N1, harness host cells' ability to replicate during respiratory illness found that the expression of *CWC22* was linked with a decreased cellular infection rate (52). How these genes might be influencing BCoV infection in cattle is unclear, but the overlap with other coronavirus studies and pulmonary health factors suggest the connections are worth further investigation.

Little is known about the functions of the last five positional candidate genes associated with BCoV^+^BRD^+^ infection in two of the cattle populations. For example, *LOC100849043* and *LOC104969525* are listed as uncharacterized genes in cattle. After comparing the bovine sequences for *LOC100849043* and *LOC104969525* to the nucleotide sequences of other species, the only similarities with this sequence in other species were identified in water buffalo, bison, and sheep. In these species, as in cattle, the gene was uncharacterized but predicted to be a non-coding RNA. The serine protease 48 (*PRSS48*) and SH3 domain-containing 19 (*SH3D19*) genes do not have known functions relating to viral infections or respiratory illness. However, studies have shown that *PRSS48* and *SH3D19* have roles in disease (53, 54). For example, a previous study in brain endothelial cells exposed to *Cryptococcus neoformans* indicated that infection caused expression differences in *PRSS48* (53), suggesting a potential role in endothelial cells during infection. This is in keeping with a recent study that compared bronchi alveolar lavage fluid of healthy patients and patients infected with SARS-CoV2. Zhou et al. (54) reported that *SH3D19* was part of a network of genes that was differentially expressed in SARS-CoV2 patients. Similar to *PRSS48*, A-kinase anchoring protein 9 (*AKAP9*) has functions relating to endothelial cells. Sehrawat et al. (55) reported that *AKAP9* is capable of directly regulating *EPAC1*, which is involved in the regulation of the inflammatory response within umbilical and dermal endothelial cells. How this gene might function within pulmonary endothelial cells has not been investigated.

Several positional candidate genes were associated with GPCR signaling pathways (*AKAP9, PRKCA*, and *WWOX*). GPCR signaling is involved in a plethora of physiological processes, including immune responses (56). Within the immune system, GPCR signaling pathways are associated with vascular inflammation, through the promotion of proinflammatory cytokine signaling and through the disruption of the endothelial barrier in smooth muscles (57). Limited research is available on the role of GPCR in BRD or other lung infections in cattle. However, a previous study, utilizing the same cattle populations as this study, investigated gene sets associated with BRD susceptibility (22). Neupane et al. found that within the feedlot population, the regulation of a G protein-coupled receptor signaling pathway was enriched (normalized enrichment score = 3.07) for BRD susceptibility. There is considerably more knowledge on the function of GPCR signaling pathways with other coronaviruses. For example, a recent study indicated that SARS-CoV-2 may hijack the GPCR signaling pathway to alter fluid and ion transport within the lungs, which leads to lung edema—a deadly clinical sign of COVID-19 infection (58). Similarly, Hammoudeh et al. (59) suggested that SARS-CoV-2 manipulation of the GPCR pathways could facilitate viral infection within the host leading to dysregulation of intracellular transport within the livers of infected patients. Given its role in infectivity of other coronaviruses, further investigation into GPCR signaling and its association with BCoV and BRD in cattle is warranted.

In conclusion, the identification of loci associated with BCoV+ as well as BCoV+BRD+ could allow for the future selection of cattle with decreased susceptibility to both BCoV infection and BRD. Given the high economic cost as well as the animal welfare issues associated with both diseases, selecting for cattle that are less likely to be infected with BCoV and BRD is advantageous. Determining how positional candidate genes could be influencing the disease process is also of importance as coronaviruses become more prevalent worldwide in cattle and may be helpful in understanding the disease process of other coronaviruses. Possible shared mechanism across species could provide additional insight into the disease etiology and potentially future treatment options.

## Data Availability Statement

The datasets presented in this study can be found in online repositories. The names of the repository/repositories and accession number(s) can be found at: CattleQTLdb : https://www.animalgenome.org/QTLdb/supp/?t=QzYj1V9CmR.

## Ethics Statement

The animal study was reviewed and approved by Institutional Animal Care and Use Committees from—Washington State University, University of California—Davis, New Mexico State University, and Texas A&M University. Written informed consent for participation was not obtained from the owners because the need for written consent forms from each dairy and feedlot was deemed unnecessary according to the Washington State University Institutional Review Board 45 CFR 46.102(e) (1). However, each dairy and feedlot provided written agreement to participate in the study.

## Author Contributions

HN designed the study, collected the samples, reviewed, and edited the manuscript. JK performed the experiments, wrote, and edited the manuscript. All the authors read and approved the final manuscript.

## Conflict of Interest

The authors declare that the research was conducted in the absence of any commercial or financial relationships that could be construed as a potential conflict of interest.

## Publisher's Note

All claims expressed in this article are solely those of the authors and do not necessarily represent those of their affiliated organizations, or those of the publisher, the editors and the reviewers. Any product that may be evaluated in this article, or claim that may be made by its manufacturer, is not guaranteed or endorsed by the publisher.

## Footnotes

^1^Golden Helix, Inc. SNP & Variation Suite (Version 8.). Available online at: http://www.goldenhelix.com.

## Supplementary Material

Supplementary Figure 1 Example of the McGuirk scoring system used to classify cattle as bovine respiratory disease cases and controls.

Supplementary Table 1 Significant loci associated with bovine coronavirus infection in a dairy, a feedlot, and a combined dairy and feedlot population.

Supplementary Table 2 Significant loci associated with bovine coronavirus and bovine respiratory disease infections in a dairy, a feedlot, and a combined dairy and feedlot population.
