# Supplementary figures and images for: Identifying Loci Associated With Bovine Corona Virus Infection and Bovine Respiratory Disease in Dairy and Feedlot Cattle
Source: Front Vet Sci. 2021 Aug 2;8:679074. doi: 10.3389/fvets.2021.679074 (PMC8364960; doi:10.3389/fvets.2021.679074)

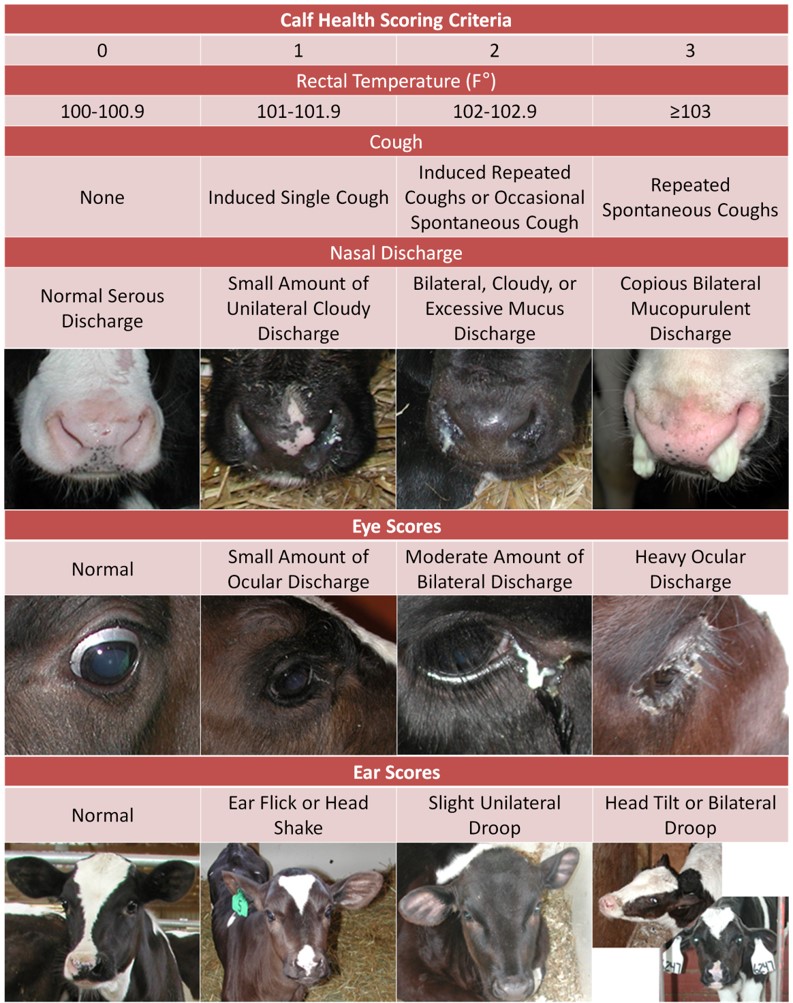

Supplement: Supplementary Figure 1 — Example of the McGuirk scoring system used to classify cattle as bovine respiratory disease cases and controls. [file Image_1.JPEG]
