# Supplementary material for: Identifying Loci Associated With Bovine Corona Virus Infection and Bovine Respiratory Disease in Dairy and Feedlot Cattle
Source: Front Vet Sci. 2021 Aug 2;8:679074. doi: 10.3389/fvets.2021.679074 (PMC8364960; doi:10.3389/fvets.2021.679074)
Supplement: Supplementary Table 1 — Significant loci associated with bovine coronavirus infection in a dairy, a feedlot, and a combined dairy and feedlot population. [file Table_1.DOCX]

**Supplementary Table 1.** Significant loci associated with bovine coronavirus infection in a dairy, a feedlot, and a combined dairy and feedlot population.

| **BTA^1^** | **BP Position** | **SNP ID^2^** | **Population(s)^3^** | **Model(s)** | **P-Valu**e | **FDR^4^** | **PCG(s)^5^** |
| --- | --- | --- | --- | --- | --- | --- | --- |
| 1 | 13,711,980 | *rs134092093* | D | Dominant | 6.84 × 10^-6^ | 0.8791 | *-* |
| 1 | 13,717,023 | *rs137526342* | D | Dominant | 4.44 × 10^-6^ | 0.9508 | *-* |
| 1 | 17,835,385 | *rs135537615* | D | Recessive | 3.59 × 10^-6^ | 0.3844 | *-* |
| 1 | 102,993,521 | *rs136327364* | F | Recessive | 3.44 × 10^-6^ | 0.2764 | *-* |
| 2 | 18,177,716 | *rs43294617* | D | Recessive | 7.21 × 10^-6^ | 0.4635 | ***TTN*** |
| 2 | 18,178,363 | *rs43294618* | D | Recessive | 3.59 × 10^-6^ | 0.3844 | ***TTN*** |
| 2 | 18,207,238 | *rs43295583* | D | Recessive | 7.21 × 10^-6^ | 0.4214 | ***TTN*** |
| 2 | 18,216,415 | *rs43295569* | D | Recessive | 7.21 × 10^-6^ | 0.3863 | ***TTN,*** *LOC112442546* |
| 2 | 56,329,269 | *rs134202902* | D | Additive  Dominant | 8.37 × 10^-6^  8.37 × 10^-6^ | 1.0000  0.5978 | *-* |
| 2 | 66,371,077 | *rs109396116* | C | Dominant | 5.03 × 10^-6^ | 1 | *-* |
| 2 | 97,266,841 | *rs134449070* | F | Recessive | 9.62 × 10^-6^ | 0.2574 | ***MAP2*** |
| 3 | 7,176,420 | *rs136025967* | C | Recessive | 4.76 × 10^-6^ | 0.3820 | ***-*** |
| 3 | 7,179,143 | *rs109554793* | C | Recessive | 4.37 × 10^-6^ | 0.4677 | ***-*** |
| 3 | 7,180,042 | *rs137421314* | C | Recessive | 5.68 × 10^-6^ | 0.3646 | ***-*** |
| 3 | 7,228,232 | *rs136625828* | C | Recessive | 4.86 × 10^-6^ | 0.3469 | ***-*** |
| 3 | 10,332,764 | *rs109115551* | F | Additive  Recessive | 6.38 × 10^-6^  3.11 × 10^-6^ | 0.5860  0.2854 | *OR10J1* |
| 3 | 97,515,856 | *rs132929867* | F | Dominant | 8.83 × 10^-6^ | 0.6303 | ***AGBL4, BEND5*** |
| 5 | 27,543,717 | *rs110585492* | F | Additive | 8.51 × 10^-6^ | 0.5469 | ***KRT89,*** *KRT85* |
| 6 | 22,884,733 | *rs41595630* | F | Recessive | 1.53 × 10^-6^ | 0.2453 | ***BANK1*** |
| 6 | 22,885,521 | *rs42572895* | F | Recessive | 8.84 × 10^-6^ | 0.3788 | ***BANK1*** |
| 7 | 71,188,419 | *rs42793197* | F | Recessive | 5.35 × 10^-7^ | 0.1146 | *-* |
| 7 | 75,632,507 | *rs41623848* | F | Dominant | 6.48 × 10^-6^ | 0.5205 | *-* |
| 8 | 46,123,575 | *rs42657192* | C | Recessive | 4.46 × 10^-6^ | 0.4095 | ***-*** |
| 10 | 770,005 | *rs135512915* | F | Recessive | 3.93 × 10^-6^ | 0.2804 | ***MCC*** |
| 10 | 15,980,581 | *rs137354456* | F | Additive | 3.35 × 10^-6^ | 0.4304 | *-* |
| 11 | 13,275,567 | *rs43667440* | F | Recessive | 7.66 × 10^-6^ | 0.3787 | *-* |
| 11 | 13,282,493 | *rs41664417* | F | Recessive | 7.66 × 10^-6^ | 0.3517 | *-* |
| 11 | 51,044,307 | *rs134621817* | D | Recessive | 5.50 × 10^-6^ | 0.5049 | *-* |
| 12 | 14,456,910 | *rs43708825* | F | Recessive | 1.56 × 10^-8^ | 0.0100 | ***SMIM2*** |
| 14 | 4,609,469 | *rs110901614* | C | Dominant | 8.16 × 10^-6^ | 1.0000 | ***FAM135B*** |
| 14 | 22,875,389 | *rs132924262* | D | Dominant | 4.90 × 10^-6^ | 0.7869 | ***XKR4*** |
| 14 | 78,204,750 | *rs136847588* | F | Recessive | 6.09 × 10^-6^ | 0.3262 | ***RALYL*** |
| 16 | 19,769,162 | *rs110829272* | C | Recessive | 6.83 × 10^-6^ | 0.3991 | ***-*** |
| 18 | 1,104,644 | *rs137656041* | F | Additive  Recessive | 1.53 × 10^-7^  3.36 × 10^-8^ | 0.0981  0.0108 | *-* |
| 18 | 3,803,365 | *rs134887854* | F | Dominant | 5.73 × 10^-7^ | 0.1227 | *-* |
| 18 | 3,804,079 | *rs135784581* | F | Additive  Dominant | 3.06 × 10^-6^  7.88 × 10^-8^ | 0.4922  0.0506 | *-* |
| 18 | 3,805,769 | *rs135201977* | F | Dominant | 2.39 × 10^-6^ | 0.2562 | *-* |
| 18 | 3,806,882 | *rs132905376* | F | Dominant | 1.84 × 10^-7^ | 0.0592 | *-* |
| 18 | 3,833,849 | *rs137156349* | F | Dominant | 9.13 × 10^-7^ | 0.1467 | *LOC112442251* |
| 18 | 5,420,736 | *rs109736099* | F | Recessive | 2.26 × 10^-6^ | 0.2910 | ***WWOX*** |
| 18 | 5,745,953 | *rs110552632* | C | Recessive | 2.50 × 10^-6^ | 0.8021 | *-* |
| 18 | 5,749,984 | *rs41648932* | C | Recessive | 2.50 × 10^-6^ | 0.5347 | *-* |
| 18 | 6,238,217 | *rs110415131* | D | Recessive | 2.17 × 10^-6^ | 0.2787 | ***MAF*** |
| 18 | 16,846,809 | *rs109849867* | D | Additive  Dominant | 1.95 × 10^-7^  1.95 × 10^-7^ | 0.1251  0.1251 | *-* |
| 19 | 7,610,664 | *rs110881085* | D | Recessive | 8.58 × 10^-6^ | 0.4243 | ***C19H17orf67,*** *LOC101904010* |
| 19 | 8,294,507 | *rs41901998* | D | Recessive | 8.35 × 10^-7^ | 0.1789 | ***MSI2*** |
| 19 | 8,325,972 | *rs134736482* | D | Recessive | 5.09 × 10^-7^ | 0.3268 | ***MSI2*** |
| 19 | 8,360,868 | *rs133988665* | C  D | Recessive  Recessive | 5.93 × 10^-7^  5.09 × 10^-7^ | 0.3810  0.1634 | ***MSI2*** |
| 19 | 16,331,543 | *rs41902948* | C | Recessive | 2.55 × 10^-6^ | 0.4097 | ***-*** |
| 19 | 62,757,064 | *rs41926236* | F | Recessive | 4.68 × 10^-6^ | 0.3008 | ***PRKCA*** |
| 19 | 62,763,041 | *rs41926279* | C | Recessive | 2.63 × 10^-6^ | 0.3380 | ***-*** |
| 20 | 12,619,125 | *rs41618472* | F | Recessive | 5.39 × 10^-6^ | 0.3148 | ***MAST4,*** *LOC112442988* |
| 20 | 13,193,930 | *rs135905544* | F | Additive  Dominant | 5.57 × 10^-7^  2.60 × 10^-6^ | 0.1791  0.2390 | *-* |
| 20 | 13,197,693 | *rs133650515* | F | Additive  Dominant | 2.05 × 10^-6^  2.28 × 10^-6^ | 0.4396  0.2932 | *-* |
| 20 | 71,896,191 | BovineHD3300001219 | D | Additive  Dominant | 6.90 × 10^-6^  6.90 × 10^-6^ | 1  0.7393 | ***IGHG1*** |
| 21 | 29,724,236 | *rs136460457* | D | Recessive | 1.24 × 10^-6^ | 0.1988 | ***LOC789928,*** *LOC100140346* |
| 21 | 35,788,704 | *rs42778981* | F | Recessive | 9.87 × 10^-6^ | 0.2536 | *-* |
| 21 | 59,923,659 | *rs110163614* | D | Additive  Dominant | 4.95 × 10^-6^  8.17 × 10^-6^ | 1  0.6564 | *-* |
| 21 | 61,261,616 | *rs134210640* | F | Recessive | 8.86 × 10^-6^ | 0.3559 | ***GSKIP*** |
| 22 | 34,281,093 | *rs137802274* | D | Dominant | 7.78 × 10^-6^ | 0.7137 | *-* |
| 22 | 43,345,133 | *rs42010826* | F | Recessive | 9.36 × 10^-6^ | 0.3539 | ***SLMAP*** |
| 22 | 43,385,429 | *rs43767373* | F | Recessive | 9.36 × 10^-6^ | 0.3342 | ***SLMAP,*** *LOC782021* |
| 22 | 43,399,874 | *rs42010793* | F | Recessive | 9.36 × 10^-6^ | 0.3166 | ***SLMAP,*** *LOC782021* |
| 22 | 43,444,760 | *rs42010785* | F | Recessive | 9.36 × 10^-6^ | 0.3008 | ***SLMAP*** |
| 22 | 43,453,747 | *rs109896564* | F | Recessive | 9.36 × 10^-6^ | 0.2865 | ***SLMAP*** |
| 22 | 43,483,997 | *rs42010765* | F | Recessive | 9.36 × 10^-6^ | 0.2735 | *SLMAP* |
| 22 | 43,488,614 | *rs42010770* | F | Recessive | 9.36 × 10^-6^ | 0.2616 | *-* |
| 22 | 46,579,224 | *rs133326900* | C | Dominant | 1.57 × 10^-6^ | 1 | ***CACNA2D3*** |
| 24 | 34,214,145 | *rs110526946* | F | Recessive | 2.50 × 10^-6^ | 0.2674 | *-* |
| 26 | 14,174,844 | *rs42808752* | C | Additive  Dominant | 9.25 × 10^-6^  2.69 × 10^-6^ | 1  0.8648 | *-* |
| 26 | 45,589,462 | *rs42441238* | D | Additive  Dominant | 2.56 × 10^-6^  2.56 × 10^-6^ | 0.8218  0.8218 | ***ADAM12*** |
| 28 | 12,279,420 | *rs110459179* | D | Recessive | 6.18 × 10^-6^ | 0.4966 | *-* |
| 28 | 12,294,261 | *rs109495685* | D | Recessive | 6.18 × 10^-6^ | 0.4414 | *-* |
| 29 | 16,313,656 | *rs109606853* | F | Additive | 6.51 × 10^-6^ | 0.5225 | *-* |
| 29 | 16,332,176 | *rs109636356* | F | Additive | 7.35 × 10^-6^ | 0.5247 | *-* |
| 29 | 16,345,120 | *rs136108464* | F | Additive | 5.65 × 10^-6^ | 0.6052 | *-* |

^1^Single nucleotide polymorphism (SNP) location as measured by numbered nucleotides in reference to the ARS 1.2 genome assembly (https://www.animalgenome.org/repository/cattle/UMC_bovine_coordinates/; accessed 21, January 2020).

^2^Each SNP is identified by *rs* number which is a reference number assigned to markers submitted to the National Center for Biotechnology Information SNP database. If no *rs* number is available, the SNP name from the Illumina BovineHD BeadChip is listed.

^3^Populations abbreviated as follows: C = combined feedlot and dairy; F = feedlot; D = dairy.

^4^False discovery rate (FDR).

^5^Positional candidate genes (PCGs) were defined as genes located within either 18 kb (dairy population), 12 kb (feedlot population) or 15 kb (combined population) on either side of the associated SNP(s). Bolded gene names represent genes where SNP are located within an intron of the gene.
